# Targeting WASF3 Signaling in Metastatic Cancer

**DOI:** 10.3390/ijms22020836

**Published:** 2021-01-15

**Authors:** Reid Loveless, Yong Teng

**Affiliations:** 1 Department of Oral Biology and Diagnostic Sciences, Dental College of Georgia, Augusta University, Augusta, GA 30912, USA; rloveless@augusta.edu; 2 Georgia Cancer Center, Department of Biochemistry and Molecular Biology, Medical College of Georgia, Augusta University, Augusta, GA 30912, USA; 3 Department of Medical Laboratory, Imaging and Radiologic Sciences, College of Allied Health, Augusta University, Augusta, GA 30912, USA

**Keywords:** WASF3, metastasis, cancer, signaling network, drug target

## Abstract

Increasing evidence indicates that cancer metastasis is regulated by specific genetic pathways independent of those controlling tumorigenesis and cancer growth. WASF3, a Wiskott–Aldrich syndrome protein family member, appears to play a major role not only in the regulation of actin cytoskeleton dynamics but also in cancer cell invasion/metastasis. Recent studies have highlighted that WASF3 is a master regulator and acts as a pivotal scaffolding protein, bringing the various components of metastatic signaling complexes together both spatially and temporally. Herein, targeting WASF3 at the levels of transcription, protein stability, and phosphorylation holds great promise for metastasis suppression, regardless of the diverse genetic backgrounds associated with tumor development. This review focuses on the critical and distinct contributions of WASF3 in the regulation of signal pathways promoting cancer cell invasion and metastasis.

## 1. Introduction

The dispersal of cancer cells from their primary growth site to secondary growth sites in the body represents the metastatic process and the leading cause of death in cancer patients today [1]. Despite their clinical implications, the genes and mechanisms underlying this process have yet to be fully elucidated owing to their expansive influence and inherent complexity. Identifying and characterizing the lead drivers of metastasis has, therefore, remained the primary motive for many cancer research groups, including ours. 

One family of proteins, the Wiskott-Aldrich syndrome protein (WASP) family, plays an important role in cell invasion and metastasis through the activation of leading edge membrane structures. The family consists of five members that are divided into two subfamilies based on their structural similarities. The WASP subfamily consists of WASP and N-WASP; and the WASF (also referred to as WAVE) subfamily consists of WASF1, WASF2, and WASF3 [2]. For all members, phosphoactivation following extracellular stimulus (e.g., cytokines or growth factors) leads to the exposure of their *C*-terminus motifs which then interact with many factors, including the actin-related protein (Arp) 2/3 complex [2]. 

**Figure 1:**
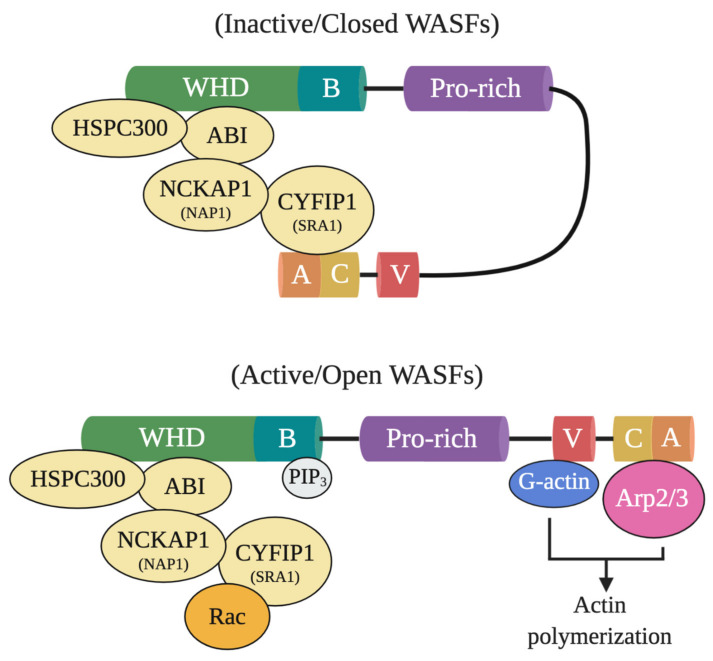
Inactive and active WASF domain confirmations. At resting state, WASFs exist in an autoinhibited closed conformational state due to the interactions between members of the *N*-terminally located WASF homology domain (WHD) and the *C*-terminus. Members of the protein complex at WHD include HSPC300, ABI, NCKAP1 (NAP1), CYFIP1 (SRA1), and WASF1/2/3. Upon activation by Rac, WHD interactions are disrupted and WASFs adopts an open conformation where their verprolin-cofilin-acidic (VCA) region can interact with the actin-related protein (Arp) 2/3 complex and monomeric actin (G-actin). Phosphatidylinositol (3,4,5)-trisphosphate (PIP3) may also recruit the WASF complex to the membrane by binding to the basic domain (B) of WASFs.

In particular, WASF members mediate the activation of Arp2/3 through a five-subunit WASF regulatory complex (WRC) which acts downstream of the GTPase Rho family member Rac [3,4]. In their normal inactive state, WASF proteins maintain an autoinhibited conformation through interactions between their *N*- and *C*-termini formed by WRC binding components, functionally blocking the verprolin-cofilin-acidic (VCA) region where the Arp2/3 complex binds (Figure 1) [5]. Following activation by Rac through constituents like the cytoplasmic FMR1 interacting protein 1 (CYFIP1, also referred to as SRA1), however, a conformational change exposes the VCA region of WASFs, allowing the CA domain to associate with the Arp2/3 complex and the V domain with monomeric actin [5]. The Arp2/3 complex then initiates actin polymerization and branched filament network formation by generating a new nucleation core and binding to pre-existing filaments [6]. From this branched nucleation process, actin projections at the leading edge of the cell (lamellipodia) and actin matrix-degrading structures (invadopodia) can be subsequently formed and used to facilitate cell motility and matrix remodeling [5]. Under normal conditions, the formation of these structures is strictly governed to safeguard tissues against disruption and damage. Under pathological conditions, however, cancer cells exploit this process to locally invade and metastasize throughout the body. 

Although each of the WASP and WASF proteins form networks of regulatory steps, of them, WASF3 represents one of the most central and critical intermediates in the signaling pathways promoting metastasis. It should be noted, however, that while WASF1 and WASF2 are not essential to invadopodium formation [7], WASF2, in particular, has attracted significant attention for its potential as a prognostic indicator and role in the invasion of cancers, like melanoma [8], breast [9], and pancreatic [10] cancers.

In normal cells, WASF3 serves in the transduction of signals leading to changes in cell morphology and cytoskeletal organization through the aforementioned regulatory mechanism and is predominately expressed in the nervous system [11]. In various human cancers, like colon [12], prostate [13], and pancreatic [14], however, *WASF3* is upregulated and, in the case of breast cancer, linked to increased invasive and metastatic cell potential through the regulation of epithelial-to-mesenchymal transition (EMT) [15,16]. Moreover, the expression of *WASF3* has been reported to be positively correlated with poorer prognosis in gastric cancer [17], non-small cell lung cancer [12], and hepatocellular carcinoma patients [18] and to be enriched in triple-negative breast cancer stem cells where it promotes self-renewal and may contribute to chemoresistance [19]. Additionally, the protein has been found to promote the invasion of ovarian cancer cells and to play an important role in their secretion of the pluripotent transcription factors Oct4 and SOX2 [20]. Lastly, WASF3 has also been linked to the regulation of matrix metalloproteinase (MMP) production, providing invading cells a means to remodel the extracellular matrix [21]. In this review, we focus our attention on the role of WASF3 in cancer metastasis by dissecting its underlying signaling network and molecular regulations.

## 2. Genetic Dissection of WASF3-Related Signal Transduction Pathways

### 2.1. Regulation of WASF3 Transcription

#### 2.1.1. Regulation by Signal Transducer and Activator of Transcription 3 (STAT3)

**Figure 2:**
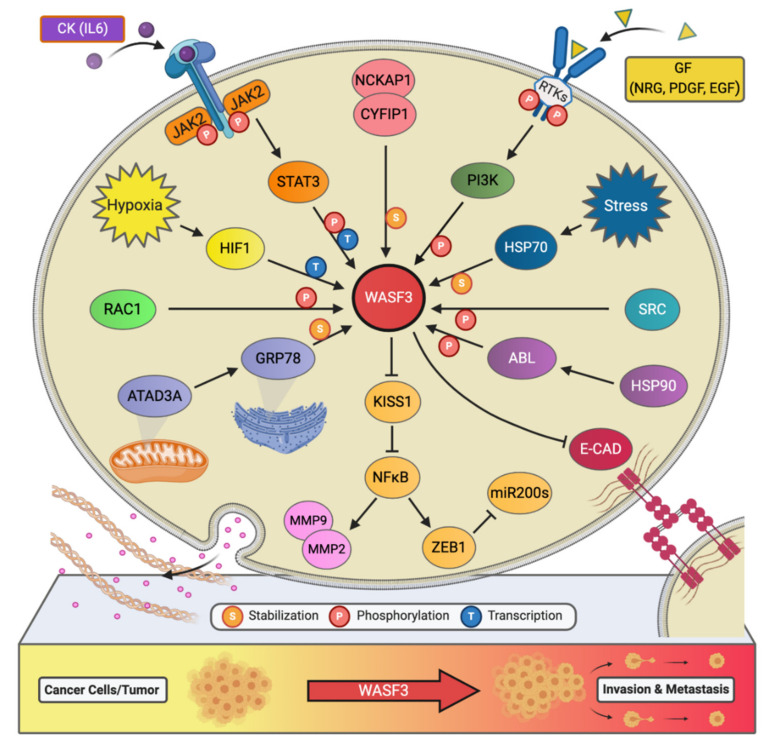
The signaling network of WASF3 in cancer metastasis. The WASF3-dependent signaling pathways and related regulatory networks critical to controlling cancer metastasis are summarized here.

Little had been revealed regarding the regulation of *WASF3* at the transcription level, however, our experiments have shown that there are three potential binding sites for STAT3 within 1100 base pairs upstream of the WASF3 transcription initiation site [22]. These sites are located at the positions -894 to -886, -915 to -906, and -926 to -919. Interleukin 6 (IL-6) is one of the many cytokines used by tumor cells to facilitate invasion and metastasis. Our results showed that within 2 min of application, IL-6 can transition WASF3 from its inactive, non-phosphorylated form in the cytoplasm to its active, phosphorylated form, which relocates to the cytoplasmic membrane and engages with Janus kinase 2 (JAK2) (Figure 2). An increase in *WASF3* gene transcription is seen to follow. Specifically, STAT3 is phosphoactivated by JAK2 kinase and relocates to the nucleus to bind to the previously mentioned binding sites to increase the transcription of *WASF3* [22]. Inhibiting the activation of STAT3 by its inhibitor S3I-201 leads to reduced *WASF3* levels, even with the application of IL-6. Although there are many receptors, this activation process was found to be largely achieved through the binding of IL-6 to the GP130 receptor. Upon blocking GP130 with BR-3 monoclonal antibody, WASF3 levels showed no change in the presence of IL-6. It was also found that JAK2 phosphoactivated WASF3 protein and that inactivation of JAK2 by the JAK kinase inhibitor AG490 led to a reduction in activated WASF3 levels [22].

#### 2.1.2. Regulation by Hypoxia-Inducible Factor 1-Alpha (HIF1A)

It is well known that hypoxia affects the ability of cancer cells to invade and metastasize through changes in molecular pathways. Constituents of these pathways, therefore, represent potential therapeutic targets for preventing metastasis. In particular, WASF3 is a hypoxia-inducible gene, meaning that it contains at least one or more hypoxia responsive elements (HRE) which act as binding sites for transcription factors [23]. The levels of WASF3 protein increased significantly in MDA-MB-231 and MCF7 breast cancer cells incubated under hypoxic conditions. In the promoter region of the *WASF3* gene, four HREs were identified. HRE1, 2, and 3 are located in between -27 and -79 base pairs of the first exon of the *WASF3* gene, and HRE4 is located distally around -721 and -725 base pairs to the same reference point. HIF1s bind to these HRE and increase the transcription of *WASF3* in a hypoxic environment. The HIF1A factor is required to facilitate this increment change in *WASF3* levels, as knockdown of HIF1A, but not HIF2A, was found to reduce the levels of *WASF3* in cells undergoing hypoxia (Figure 2) [23]. The phosphoactivation process of WASF3 was also increased under hypoxic conditions, as well as the levels of MMP1, MMP3, and MMP9. One study showed that combining anti-HIF (YC-1) and anti-VEGF (SU5416) treatments in MDA-MB-231 cells under these conditions decreases *WASF3* levels dramatically in comparison to anti-VEGF treatment alone [23]. The possible mechanism behind this observation is that inhibition of VEGF may target the angiogenic ability of cancer cells and create a hypoxic environment that, in turn, increases *WASF3* levels. The use of conventional anti-angiogenic cancer treatments that rely solely on this type of medication, therefore, might need to be reconsidered.

### 2.2. Regulation of WASF3 Protein Stability

#### 2.2.1. Regulation by Heat Shock Protein 70 (HSP70)

Chaperone proteins play critical roles in facilitating client protein activation and/or maintaining their stability. Examples of chaperone proteins include HSP90 and HSP70. While HSP90 has been linked to WASF3 activation through ABL kinase, HSP70 has been seen to preserve WASF3 protein stability in the cytoplasm and to protect it from proteasome degradation [24]. Using MDA-MB-231 cells expressing a series of truncated, HA-tagged WASF3 constructs, HSP70 interaction with WASF3 was found to take place at WASF3’s *N*-terminus (Figure 2). Interfering with the transcriptional activation of *HSP70* with KNK437 in HEK-293 cells also led to a reduction in the protein levels of both HSP70 and WASF3 while leaving *WASF3* mRNA levels unchanged. Accordingly, heat shock experiments across various cancer cell lines further resulted in increased levels of HSP70 and WASF3 protein without an increase in *WASF3* mRNA [24].

Given that HSP70 binding frequently functions to target client proteins for degradation, treatment with the proteasome inhibitor MG132 alone or in combination with HSP70 inhibitors provided further clarification into the HSP70-mediated stabilization of WASF3. Specifically, it was found that disrupting the association of HSP70 and WASF3 using PEP, even in the presence of MG132, led to a reduction in WASF3 protein levels [24]. This suggests that it is the formation of the HSP70-WASF3 complex, rather than endogenous HSP70 protection from proteasome degradation that provides WASF3 stability. Further study also revealed that silencing or pharmacological inhibition of HSP70 hinders cell migration while overexpressing it elevates WASF3 protein levels and invasion [24]. Aside, others have reported that an HSP70/WASF3/MMP9 axis can be further upregulated to promote breast cancer cell invasion through p63α, a member of the p53 family that is critical to HSP70 expression [25]. Taken together, these findings contribute to the basis of our understanding of HSP70’s importance in WASF3 stabilization and, in turn, WASF3-mediated cancer cell invasion and metastasis. 

#### 2.2.2. Regulation by ATAD3A-Dependent GRP78 

AAA domain containing 3A (ATAD3A) is a ubiquitously expressed mitochondrial membrane ATPase that contributes to the regulation of mitochondrial dynamics [26]. Recently, ATAD3A has been the subject of intense interrogation for its role throughout cancer progression and, more specifically, its role in regulating WASF3 facilitated metastasis [27,28]. In engraftment experiments using MDA-MB-231 cells and immunocompromised mice, knockdown of ATAD3A markedly suppressed tumor growth, neovascularization, and the incidence of metastatic colonies (Figure 2). In vitro, *ATAD3A* silencing was also found to significantly reduce WASF3 protein levels while leaving *WASF3* mRNA levels unchanged. Importantly, this silencing was additionally accompanied by a decrease in cell anchorage-independent growth and invasion [28].

While HSP70 serves to stabilize WASF3 in the cytoplasm, it is presumed that ATAD3A, alongside GRP78, maintain this role in the mitochondria. In particular, inactivation of HSP70 was seen to significantly reduce the levels of cytoplasmic WASF3 but not the levels of mitochondrial-associated WASF3. Immunofluorescent signaling representing WASF3-mitochondria association, however, was seen to be entirely diminished following *ATAD3A* knockdown. Through ATAD3A truncation experiments, it was additionally observed that WASF3 interacts exclusively with the *N*-terminus of ATAD3A and that this interaction is not only crucial to WASF3 stabilization but independent of HSP70. Aside, the *N*-terminus of WASF3 was found to penetrate the outer mitochondrial membrane, which protects against proteolysis. Interestingly, the endoplasmic reticulum (ER) resident chaperone protein GRP78 was also found to be implicated in ATAD3A-mediated WASF3 stabilization (Figure 2). Under normal conditions, GRP78 contributes to the coordination of the unfolded protein response following ER stress [28].

In both breast and colon cancer cell lines, it was observed that while *ATAD3A* knockdown decreased the protein levels of GRP78 and WASF3, WASF3 levels could be ameliorated by overexpressing *GRP78*. Moreover, in cells overexpressing *WASF3*, the half-life of WASF3 could be significantly extended if GRP78 was also overexpressed. Accordingly, *GRP78* knockdown did not affect either the protein levels of ATADA3 or gene expression of *WASF3* but did reduce the levels of WASF3 protein. Lastly, ATAD3A-GRP78 binding was also observed between ER and mitochondrial membranes and, under ER stress, it was seen that WASF3 stabilization was enhanced through increased WASF3-GRP78 interaction [28]. ATAD3A stabilization of WASF3, therefore, is likely dependent on GRP78. Further study in our group reveals that ATAD3A suppresses CDH1/E-cadherin expression through its regulation of WASF3 that is associated with GRP78 function [28].

### 2.3. Regulation of WASF3 Protein Phosphorylation 

#### 2.3.1. Regulation by Phosphoinositide 3-Kinase (PI3K)

In addition to IL-6, platelet-derived growth factor (PDGF) has also been found to influence the activity of the WASF3 protein [29]. In particular, following MDA-MB-231 cell treatment with PDGF, it was discovered that interaction between WASF3 and PI3K is required for lamellipodia formation and cell migration. Structurally, the PI3K protein consists of two subunits, a p110 catalytic subunit and a p85 regulatory subunit which is an important effector for actin cytoskeleton remodeling and, thus, cell migration. Upon PDGF binding, PI3K is recruited to the activated PDGFR and binds to it through its p85 *C*-terminus SRC homology 2 (SH2) domain, resulting in internalization of the complex into the cytoplasm. Interestingly, it was found that WASF3 also interacts with PI3K at this same SH2 domain and that treating cells with the PI3K inhibitor LY294002 significantly decreases lamellipodia formation and cell migration, suggesting that WASF3-p85 interaction may play a part in actin polymerization and cell invasion (Figure 2) [29]. 

In another study, pharmacological inhibition of PI3K isoforms was similarly found to decrease cell motility, as well as suppress *WASF3* induction in TLR5/7-treated ovarian SK-OV-3 cancer cells [20]. It was also revealed that following the PI3K-mediated activation of WASF3, WASF3 goes on to play a critical role in the expression of mesothelin and production of Oct4/SOX2 in TLR5/7-mediated SK-OV-3 cell invasion [20]. Aside, it has recently been reported that WASF3 phosphorylation acts to modulate a positive feedback loop between WASF3 and the PI3K-TGF-β-EGF signaling pathways [16], which supports previous findings that depletion of *WASF3* expression in breast cancer cells prevents TGF-β-mediated EMT and lamellipodia formation in metastatic cells [30]. 

#### 2.3.2. Regulation by JAK2

Interestingly, an increase in phosphoactivated WASF3 and WASF3 protein levels was observed in prostate cancer DU145 cells following the treatment of IL-6 [22]. The same tendency was also seen in breast cancer MDA-MB-231 cells after IL-6 stimulation [22]. The cells receiving the pan JAK inhibitor AG490 showed no activation of WASF3, while those receiving the STAT3 inhibitor S31-201 showed reduced levels of activated WASF3 owing to a decrease in the total amount of WASF3 [22]. To dig deeper into which of the JAK members were implicated, we knocked down either *JAK1* or *JAK2* in MDA-MB-231 cells and subsequently found a significant reduction in WASF3 levels in *JAK2* knockdown cells but not JAK1 [22]. From these findings, we concluded that IL-6 induces WASF3 phosphoactivation through the JAK2 pathway, which facilitates WASF3’s relocation to the cell membrane and the reorganization of the intracellular actin cytoskeleton, leading to cell migration and metastasis.

#### 2.3.3. Regulation by Abelson Tyrosine Kinase (ABL)

Another critical component to WASF3’s phosphoactivation is ABL, whose presence in the WASF3 immunocomplex was confirmed through immunoprecipitation (IP) analysis of MDA-MB-231 cells treated with PDGF. In one study, treatment of MDA-MB-231 cells with the ABL kinase inhibitor Gleevec (STI-571) led to a dramatic decrease in WASF3 phosphorylation levels [11]. Moreover, four tyrosine residues in the amino acid sequence of WASF3 were identified as possible targets of ABL mediated phosphorylation, including Tyr-151, Tyr-248, Tyr-337, and Tyr-486. Only when all four of these residues are mutated does ABL kinase lose its ability to carry out its role in this phosphorylation process and lamellipodia formation and cell invasion decrease [11].

#### 2.3.4. Regulation by HSP90

Chaperone proteins like HSP90 are necessary for the proper folding and degradation of client proteins, affecting their stability and levels inside the cell. When PC3, MDA-MB-231, SKBR3, and COS7 cell lines were treated with the HSP90 inhibitor 17-AAG that acts by binding to the ATP/ADP binding pocket on HSP90’s *N*-terminus, there were no observable changes in WASF3 protein levels [24]. Similar results were also seen with the treatment of Novobiocin, another HSP90 inhibitor that instead binds at HSP90’s *C*-terminal nucleotide-binding pocket, suggesting that WASF3 is likely not a client protein for HSP90. 

Treatment of PC3 cells with 17-AAG for 24-h did, however, lead to markedly decreased WASF3 phosphoactivation and ABL levels, even in the presence of PDGF. To investigate the interaction between HSP90 and ABL, IP, and Western blot analysis of PC3 cells was performed using anti-ABL antibody and confirmed the presence of HSP90 in the complex. Importantly, when 17-AAG was used to inhibit HSP90, ABL levels were undetectable. Taken together, these results indicate that ABL is a client protein of HSP90 and that through HSP90 targeting, WASF3 phosphoactivation is inhibited as a result of ABL kinase destabilization, leading to a decrease in cell invasion and metastasis potential [24].

#### 2.3.5. Regulation by Human Epidermal Growth Factor Receptor 2/3 (HER2/HER3) Signaling Axis 

HER2 (ERBB2/neu) and HER3 (ERBB3) are receptor tyrosine kinases implicated in cancer invasion and metastasis. Of these two, *HER2* is overexpressed in 20–30% of breast cancers and is strongly associated with poor patient prognosis [31]. While HER3 has also been linked to worse cancer patient outcomes, it contrasts with its family members by lacking tyrosine kinase activity [32]. Through heterodimerization with, for example, HER2, however, HER3 can contribute to signaling activation [32]. In fact, our studies have shown that WASF3-mediated cancer invasion is contingent upon the HER2/HER3 heterodimer, which facilitates WASF3 phosphoactivation and transcription [33]. Specifically, the functional activation of HER2 using NRG was seen to induce the phosphorylation of WASF3 in HER2-positive SKBR3 breast cancer cells. Through confocal microscopy and IP, visualization of WASF3 recruitment to the membrane and interaction with HER2, as well as HER2’s presence in the WASF3 immunocomplex, was also achieved.

After investigating the expression profile and phosphorylation status of HER2 and HER3, it was found that, unlike HER2, HER3’s activation and presence in the WASF3 immunocomplex were contingent upon NRG treatment. Moreover, using siRNA knockdown, it was demonstrated that WASF3 phosphorylation required the expression of both *HER2* and *HER3*, which suggests a key role for HER2/HER3 heterodimerization in WASF3 activation. Blocking HER2 binding using Herceptin also significantly inhibited WASF3 phosphorylation and hindered NRG-induced invasion in *WASF3* overexpressing cells. Importantly, this observation was not seen with the treatment of Erlotinib, allowing the influence of EGFR to be excluded. Furthermore, unlike the long-term treatment of NRG which resulted in a four-fold increase in WASF3 protein levels, EGFP treatment did not affect WASF3 levels. These findings suggested that NRG and HER2 are involved in the transcriptional regulation of *WASF3*, which RT-PCR later confirmed, showing the mRNA levels of both *WASF3* and *WASF1* to be increased after long-term exposure to NRG [33].

In previous studies, we have also identified STAT3 to be a key regulator of WASF3 expression. Again using SKBR3 cells, we found that NRG stimulation significantly increases STAT3 phosphorylation and that blocking the activation of either STAT3 or JAK2 prevents NRG-induction of WASF3, indicating that WASF3 activation is dependent on HER2/HER3-JAK2/STAT3 signaling [33]. Finally, invasion and metastasis were also evaluated in vitro and in vivo using MCF7 cells and mice and were found to be significantly enhanced following the co-expression of *WASF3* and *HER2*, further demonstrating the crucial role of HER2-WASF3 in cancer metastasis [33].

## 3. Controlling Invasion and Metastasis through WASF3 and microRNA (miRNA or miR) Interactions

### 3.1. Regulation by chr1-miR-200s

miRNAs are non-coding sequences of nucleotides whose increased or decreased expression has been linked to the metastatic and invasive phenotypes of cancer cells [34]. miR-10b, 373, and 520c are examples of metastasis promoting miRNAs and are seen to be upregulated in *WASF3* overexpressing T47D breast cancer cells [15]. Members of the miRNA-200 family have also been implicated in cancer invasion through their regulation of EMT and include miR-200a, miR-200b, and miR-429 clustered on chromosome 1 (called Chr1-miR-200s) and miR-200c and miR-141 clustered on chromosome 12. In one study, it was found that miRNA-200 levels were inversely correlated with *WASF3* levels in cancer cells and that miRNA-200b inhibits *WASF3* expression by directly targeting the 3′- untranslated regions (UTR) of *WASF3* mRNA [35]. Interestingly, it was reported that this inhibition was specific to WASF3 and not its other family members and that deletion of the target motifs within *WASF3*’s 3′-UTR abrogated the effect of exogenous and endogenous miR-200s (Figure 2). Following the introduction of an anti-miRNA-200b oligonucleotide, an increase in cell invasion was also observed [35]. In large, these findings are in accordance with our experiments, where *WASF3* levels were seen to be inversely correlated with Chr1-miR-200s levels, but positively correlated with the levels of the transcription factor zinc finger E-box-binding homeobox 1 (ZEB1) [15]. By directly binding to specific sequences called E-boxes and recruiting either co-activators or co-suppressors, ZEB1 can, respectively, up- or downregulate its target genes. Notably, ZEB1 is a key player in tumor invasion and metastasis through its inducement of EMT [36]. 

In particular, the promoter of Chr1-miR-200s contains paired ZEB binding sites that, when bound by ZEB1, inhibit the promoter’s activity and decrease the levels of miR-200a, 200b, and 429. In line with this, we have shown that *ZEB1* knockdown in T47D and MCF7 cells markedly increases their expression of Chr1-miR-200s. Moreover, it was demonstrated that Chr1-miR-200s play a critical role in preventing WASF3-mediated cell invasion and are crucial to the maintenance of cell surface E-cadherin levels [15], which are typically depleted when *WASF3* is overexpressed [37]. In both T47D and MCF7 cells, the introduction of locked nucleic acid-modified oligonucleotides targeting miR-200a and miR-200b was followed by an increase in cell invasion potential; whereas the overexpression of miR-200a and miR-200b in T47D cells led to a decrease in cell invasion potential, even when *WASF3* was overexpressed [15]. 

Through additional investigation, it was further revealed that increased levels of ZEB1 and p65/50 following *WASF3* overexpression result from WASF3’s downregulation of *KISS1*, causing IκBα’s inhibition over IKKα/βto be lifted [15]. Following a decrease in IκBα levels, the nuclear translocation of p65/50 and activation of NFκB both increase, which is supported by a separate study conversely showing WASF3 loss to inhibit NFκB signaling as a result of decreased NFκB nuclear translocation [38]. NFκB then binds to the ZEB1 promoter region, where p65 is seen to directly interact with the NFκB response element, driving *ZEB1* expression. Notably, overexpression of *WASF3* in T47D cells was also seen to result in higher levels of p65 at the promoter region, suggesting a role for p65 in the regulation of ZEB1 at the transcriptional level [15]. Taken together, however, it is clear that overexpression of *WASF3* leads to an increase in the levels of ZEB1 which suppresses Chr1-miR-200s and their anti-metastatic effect. 

### 3.2. Regulation by miR-217 

miR-217 has also been associated with aggressive tumor phenotypes and poor overall survival in certain cancer types [39]. In osteosarcoma, for example, miRNA-217 expression was found to be remarkably lower in metastatic tissues than normal tissues. Moreover, treatment of MG-63 osteosarcoma cells with miR-217 mimetic is reported to significantly suppress their ability to migrate [40]. Interestingly, the complementary sequence of miR-217 has been identified in the 3′-UTR of *WASF3* mRNA, indicating that miR-217 directly targets *WASF3* expression [40]. In line with this idea, WASF3 protein levels are significantly reduced following miR-217 overexpression and, conversely, miR-217 levels reduced following *WASF3* overexpression in cells exhibiting invasion and metastasis [40]. Aside, these findings are similar to those surrounding miR-218 whose expression suppresses cancer cell migration and EMT and directly targets *WASF3* [41].

### 3.3. Regulation by miR-31 

It has been further revealed that the 3′-UTR of *WASF3* mRNA possesses an additional complementary sequence to another important miRNA member, miR-31 [42]. Similar to the previously mentioned miRNAs, miR-31 levels were found to be inversely correlated with levels of *WASF3* mRNA in metastatic cancer cells. While MDA-MB-231, MDA-MB-435, and BT549 breast cancer cell lines demonstrated high levels of *WASF3* and low levels of miR-31, the reverse was seen in non-metastatic T47D and MCF7 cell lines (low *WASF3*, high miR-31). In particular, miR-31 achieves its inhibitory effect on WASF3 through its post-transcriptional repression targeting of the 3′-UTR of *WASF3* mRNA. This was confirmed in a study showing that the overexpression of miR-31 synthetic precursors leads to a decrease in *WASF3* expression and protein levels in breast cancer cells [42]. Using a Matrigel assay, the reduction in MDA-MB-231 and LNCaP cell invasion resulting from *WASF3* siRNA targeting was also seen to be comparable to that when miR-31 was overexpressed in the cells, further grounding miR-31’s importance in suppressing WASF3-mediated cell invasion [42]. 

### 3.4. Regulation by miR-93

Recently, the analysis of metastasized, CD44^+^, patient-derived breast cancer cells engrafted into mice revealed that miR-93, as well as miR-25 and miR-106b, were downregulated [43]. In vitro, miR-93 overexpression significantly hindered breast cancer cell invasion and 3D-organoid growth, and in vivo, it suppressed their ability to metastasize to the liver. *WASF3* was identified as a functional target of miR-93 and noted to experience decreased protein levels following miR-93 overexpression. The forced expression of *WASF3* in vitro, however, was seen to override miR-93’s ability to suppress breast cancer invasion. Accordingly, metastasized CD44^+^ cancer stem cells in patient-derived xenograft mice were revealed to significantly upregulate *WASF3* mRNA and, as previously mentioned, downregulate miR-93 in comparison to primary cancer stem cells [43]. Although the exact functional relevance of miR-93’s interaction with *WASF3* is not yet clear, miR-93’s possible capacity to impede WASF3 regulation, for example, of stem cell properties, may provide a useful tool in future therapeutic development. 

## 4. The Critical Role of WASF3 in Facilitating the Invasion of Mutant RAS Expressing Cancer Cells

The transition of RAS proteins from their GDP-bound inactive form to their GTP-active form is a tightly regulated process mediated by growth factor stimulation. In cancer cells, however, mutations are frequently acquired that lead to the stabilization of the GTP-active form, resulting in overactive signaling and, in turn, invasion and metastasis. Previously, we have demonstrated that mutant expression or overexpression of *KRAS* markedly increases invasion, though that this phenotype can be attenuated through WASF3 inactivation [44]. Using non-transformed MCF10A overexpressing *KRAS^G12V^* cells, it was also found that *WASF3* knockdown significantly decreases the activation of AKT, a pro-metastatic serine/threonine kinase that is activated by PI3K downstream of RAS. In addition, WASF3 phosphorylation levels were observed to be remarkably higher when *KRAS^G12V^* was overexpressed in comparison to cells expressing normal *KRAS^G12V^* or *KRAS^WT^* [44].

To further investigate the relationship between these constituents, IP from MDA-MB-231 breast cancer cells was performed and confirmed the presence of AKT in the WASF3 immunocomplex, as well as p85, the regulatory subunit of PI3K. Similar to previous results, the knockdown of *WASF3* in this cell line significantly suppressed the levels of phosphoactivated AKT. In contrast, these levels were seen to increase following the overexpressing *KRAS^G12V^* and even further following the concurrent overexpression of *WASF3* in T47D breast cancer cells. In colon cancer cell lines, *WASF3* and *KRAS^G12V^* expression likewise increased AKT phosphorylation levels, as well as cell invasion. In vivo, *WASF3* knockdown in SW620 cells was also observed to markedly suppress their metastatic potential when injected into mice [44]. 

Particularly, the p110 subunit of PI3K is responsible for activating AKT and is catalytically suppressed when it associates with the p85 subunit. This p110-p85 interaction, however, was found to be reduced following *WASF3* and *KRAS^G12V^* overexpression and increased following *WASF3* knockdown. Taken together, these results indicate that the ability of mutant RAS to promote cell invasion is in part hinged on the WASF3-mediated dissociation of p110-p85, which enhances PI3K activity and, thus, increases AKT activation [44]. Aside, it is worth mentioning that in a separate investigation using pancreatic cancer cells, WASF3 was also found to affect the PI3K/AKT pathway. In particular, it was shown that *WASF3* knockdown suppressed the expression of pyruvate dehydrogenase kinase isoform 2 (*PDK2*) and negatively inhibited the phosphorylation of AKT at Ser 473 [13]. The expression of proteins downstream to AKT, such as EMT-related proteins and p53, were also observed to be downregulated following this absence of WASF3, further supporting WASF3’s potent and expansive influence [13]. 

## 5. Targeting WASF3-Dependent Metastatic Pathways

The therapeutic targeting potential of WASF3 is underscored by the fact that it functions at the nexus of various signaling pathways controlling metastasis. The CYFIP1-NCK associated protein 1 (NCKAP1, also referred to as NAP1) dimer, in particular, is part of the WRC which binds to the *N*-terminally located WHD of WASF3, keeping the protein in its inactive conformation (Figure 1). With its interactions with other proteins, the CYFIP1-NCKAP dimer effectively prevents actin polymerization through its regulation of WASF3’s VCA motif that binds monomeric actin and the Arp2/3 complex. 

**Figure 3:**
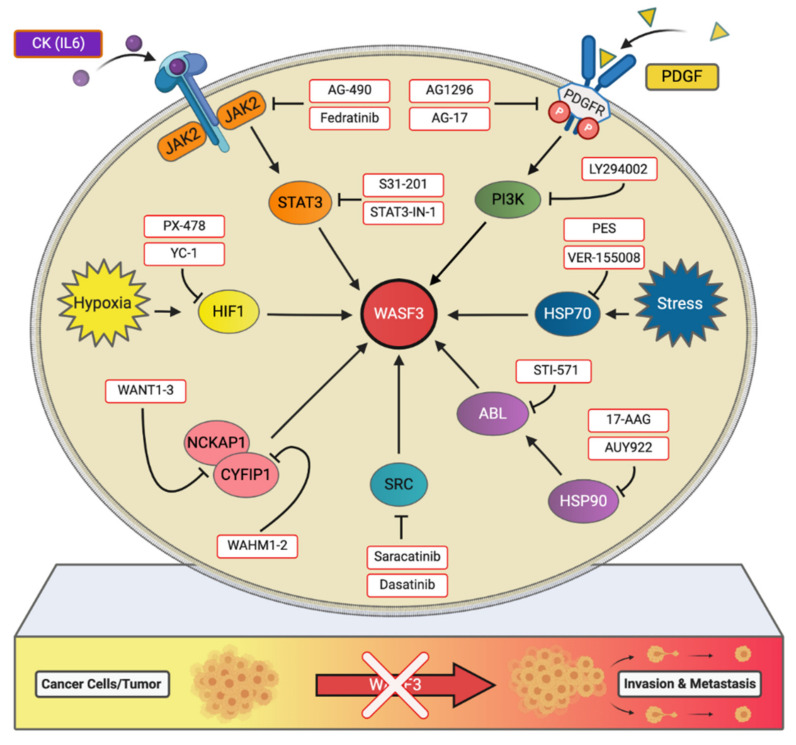
Different inhibitor strategies to abolish the activation of WASF3 signaling and suppress metastasis. Annotations in red identify nodes in this schema for which drugs are available to suppress WASF3 signaling. Only a few representative drugs are shown in each node.

Using synthetical designed stapled peptides that stabilize and constrain an α-helical structure through *N*-methylation and macrocyclic ring formation, we have previously shown that disrupting the interactions between WASF3 and CYFIP1 effectively suppresses WASF3 activation and the invasion potential of breast and prostate cancer cells (Figure 3) [45]. 

To evaluate the importance of CYFIP1 in cancer, knockdown of *CYFIP1* in breast cancer cells was performed and seen to suppress WASF3 protein levels as well as reduce cell invasion capacity [45]. While this effect was also observed across the WASF1 and WASF2 proteins, only *WASF3* knockdown resulted in a significant reduction in cell invasion in vitro and metastasis in vivo. The stapled peptides WAHM1 and WAHM2 (Figure 3), which target the α-helical interface between CYFIP1 and WASF3, were then developed and tested. Although stapled peptide treatment did not affect WASF3 protein levels, pulldown assays demonstrated the presence of CYFIP1 with both WAHM1 and WAHM2, and IP of WASF3 confirmed the absence of CYFIP1 in the immunocomplex, hinting that the peptides are capable of influencing the protein complex’s interactions or stability. Next, we evaluated how the stapled peptides affected the organization of microfilament networks in cells. In all cell lines treated with WAHM1 or WAHM2, it was observed that the actin cytoskeleton became more organized with thicker actin stress fibers, similar to that seen in *WASF3* knockdown cells [45]. A reduction in cell motility and invasion, as well as a dramatic decrease in WASF3 phosphorylation levels, were also observed. Moreover, cells treated with these stapled peptides demonstrated increased levels of KISS1 and significantly reduced levels of secreted MMP9. It was further revealed that Ras-related C3 botulinum toxin substrate 1 (Rac1) was present in the WASF3 immunocomplex, suggesting that the WAHM stapled peptides effectively interfere with the confirmation of WRC, preventing Rac1 binding and, thus WASF3 phosphoactivation and cell invasion [45]. 

The importance of NCKAP1 in WASF3-mediated invasion was also evaluated [46]. Knockdown of *NCKAP1* in cancer cells suppressed WASF3 protein levels but not *WASF3* transcript levels. Moreover, in vitro cell invasion and in vivo metastasis were markedly reduced following the knockdown of *NCKAP1*, without any demonstrated effect on proliferation. Overexpression of *NCKAP1* also increased the invasion potential of breast cancer cells, in addition to the levels of Rac1 in the WASF3 immunocomplex, suggesting NCKAP1 acts to enhances Rac1 engagement. Although analysis showed no direct contact between NCKAP1 and WASF3, we developed three stapled peptides, WANT1, 2, and 3, targeting the NCKAP1 regions interfacing CYFIP1. While no changes in invasion potential were observed following the treatment of cancer cells with either WANT1 or WANT2 (Figure 3), cells treated with WANT3 were seen to have significantly suppressed levels of invasion without any proliferative effects [46]. Through further investigation, it was found that WANT3 resulted in a dramatic decrease in WASF3 protein levels, as well as the levels of NCKAP1. Similar to WAHM stapled peptides, WANT3 cell uptake was rapid and its effect was dose-dependent, though unlike WAHM1, treatment with WANT3 was seen to be more effective in suppressing Rac1 engagement with the WASF3 complex. Nevertheless, the ability of WAHM1 and WANT3 to suppress cell invasion was found to be comparable [46]. Taken together, these proof-of-principle experiments demonstrate that WASF3’s invasive and metastatic effect is susceptible to suppression through the targeting of the protein-protein interactions critical to its stability and activation.

## 6. Conclusions

Defining the genes and/or pathways that lead to metastatic progression provides us the opportunity to develop and apply adjuvant therapies to limit the spread of cancer during its early stages. In contrast to multiple different pleiotropic events operating independently through different pathways, WASF3 is one of the few molecular determinants that control multiple aspects of the metastatic process. We and others have demonstrated that WASF3 is a master regulator of the metastasis-related phenotypes previously assigned to other proteins and that inactivating it is sufficient to suppress cancer invasion and metastasis. Delineating the mechanisms by which WASF3 gain and activation promote tumor metastasis provides us a deeper understanding of the biology of tumor progression and the tailoring needed to improve treatment regimens. Nevertheless, further investigation into WASF3’s expansive influence in metastasis is warranted. 

Historically, the invasive influence of WASF3 has been studied through manipulating its expression in cancer cells in vitro and then engrafting these cells into models, like mice and zebrafish, to track metastasis. Recently, however, a Wasf3 null mouse model introduced to the polyoma middle-T antigen (PyMT) has been successfully generated to evaluate the effects of Wasf3 loss in a spontaneous model of breast cancer metastasis [47]. Notably, the model is reported to develop palpable tumors by five weeks of age and exhibit metastasis by 4–6 months of age. Importantly, loss of Wasf3 did not appear to affect the development of the organism. While the group did report observing metastasis in the model, the number and size of metastatic lung lesions were found to be significantly reduced [47]. Consequently, the advent and potential employment of the Wasf3 deficient strain represent a new and promising opportunity to explore WASF3’s influence in cancer metastasis. 

While our understanding of WASF3 is not yet comprehensive, it appears that WASF3 acts as a pivotal scaffolding protein that brings various components of metastatic signaling complexes together temporally and spatially (Figure 2). Consequently, WASF3-targeted therapies may hold great promise for limiting metastasis regardless of the different genetic backgrounds associated with tumorigenesis. Currently, there are no effective drugs that target WASF3 directly; however, targeting the critical nodes in WASF3-driven metastatic signaling (Figure 3) is feasible, as some of these inhibitors have been discovered or already commercially available. In the future, refining stapled peptides inhibitors to interrupt WASF3-dependent immunocomplex or identifying novel *WASF3* transcript inhibitors using genome-editing tools would facilitate the development of more effective therapeutics to improve clinical outcomes in metastatic cancers.

## Data Availability

No new data were created or analyzed in this study. Data sharing is not applicable to this article.

## Abbreviations

ABL	Abelson tyrosine kinase
Arp	Actin-related protein
ATAD3A	AAA domain containing 3A
CYFIP1	cytoplasmic FMR1 interacting protein 1
EMT	epithelial-to-mesenchymal transition
ER	endoplasmic reticulum
HER2	human epidermal growth factor receptor 2
HER3	human epidermal growth factor receptor 3
HIF1A	hypoxia-inducible factor 1-alpha
HRE	hypoxia responsive element
HSP70	heat shock protein 70
HSP90	heat shock protein 70
IL-6	interleukin 6
IP	immunoprecipitation
JAK2	Janus kinase 2
miRNA or miR	microRNA
MMP	matrix metalloproteinase;
NCKAP1	NCK associated protein 1
PDGF	platelet-derived growth factor
PI3K	phosphoinositide 3-kinase
PyMT	the polyoma middle-T antigen
Rac1	Ras-related C3 botulinum toxin substrate 1
SH2	SRC homology 2
STAT3	signal transducer and activator of transcription 3
UTR	untranslated region
VCA	verprolin-cofilin-acidic
WASF3	Wiskott-Aldrich syndrome protein family member 3
WHD	WASF homology domain
WRC	WASF regulatory complex
ZEB1	zinc finger E-box-binding homeobox 1
